# Squamous Cell Carcinoma in a Burn Scar Presenting as a Necrotizing Soft Tissue Infection: A Case Report

**DOI:** 10.7759/cureus.105573

**Published:** 2026-03-20

**Authors:** Marcia A Chung, David Losier, Leonard Anderson

**Affiliations:** 1 Acute Care Surgery/Surgical Critical Care, Crouse Hospital, Syracuse, USA; 2 Surgery, Upstate University Hospital, Syracuse, USA; 3 Surgery, Wilson Medical Center, Johnson City, USA

**Keywords:** burn wounds, mu, non-grafted burn scar, skin grafting, squamous cell cancer, squamous cell neoplasm

## Abstract

Marjolin’s ulcer (MU), the most frequently encountered form of squamous cell carcinoma arising in burn scars, may manifest either acutely - within the first year post-injury - or as a chronic condition, sometimes developing decades later. It is estimated that Marjolin's ulcers represent a small percentage of all cutaneous squamous cell carcinomas originating in non-grafted burn scars that have healed by secondary intention. The early surgical grafting of burn wounds has been suggested as a potential preventive measure against malignant transformation. Marjolin’s ulcers are characteristically aggressive, often associated with poor clinical outcomes and very high recurrence rates.

We highlight a presentation mimicking necrotizing soft tissue infection in a previously grafted burn scar.

## Introduction

Marjolijn’s ulcer (MU) was first recorded by Jean-Nicolas Marjolijn in 1828. Marjolijn described it as villous changes arising from a burn scar. Despite a low incidence rate, MU presents significant clinical challenges due to its predilection for delayed diagnosis and aggressive nature.

There is typically a prolonged latency period between the initial insult and the development of MU. The latency period for developing malignancy is between two months and 72 years, with an average of 28 +/- 1.7 years [1]. The clinical presentation of MU varies. However, they characteristically arise from full-thickness skin burns that were non-grafted and allowed to heal by secondary intention [1].

MU affects people of all ages, most commonly between 40 and 60; men are more likely to develop MU than women, with a ratio of 3:1. It occurs in all race distributions [1]. MU occurs in all anatomic locations, but it is found most often in the lower and upper extremities (62%). It has also been reported in the head and neck [2].

MU has been reported to have two forms. The exophytic form is characterized by a prolonged and relatively benign course with a low chance of metastasis, and the infiltrative form is characterized by a more rapid course and elevated risk of metastatic spread. Infiltrative MU has a greater propensity to metastasize than other types of skin cancer, with metastasis to the lung, brain, liver, kidney, and distant lymph nodes commonly reported [3]. This makes its early diagnosis and treatment critical for patient outcomes.

Marjolin ulcer is a rare and aggressive form of cancer. The majority of MU arises from burn scars, but they may also result from chronic infections, traumatic wounds, wounds that were neglected or improperly treated, or other types of scars. MU is the malignant transformation of damaged skin tissue.

We present this case to highlight the diagnostic challenge of MU presenting as a necrotizing soft tissue infection.

## Case presentation

The patient is a 42-year-old Caucasian male who presented to the ER of a community hospital because of worsening pain and swelling in the left leg associated with purulent, malodorous drainage. He denied recent known trauma.

Twenty years ago, he was admitted for almost a year in a burn unit in a large level 1 trauma center and university hospital after he had sustained 85-90% second and third degree burns to his body when his motorcycle exploded when he was 26 years old. During that time, he underwent multiple operative interventions involving extensive split-thickness skin grafting, which had ultimately healed with significant associated contractures. Over the years, he developed recurrent cellulitis and drainage without ulceration in some of the grafted areas in the lower extremities and was treated and followed over the course of the past year by the local hospital-affiliated wound care center.

A few days prior to his presentation to the ER, he noticed drainage from several areas on both legs. On physical examination, he was a severely cachectic-appearing, pale man, apparent age older than stated. Vital signs were notable for a temperature of 38°C. He was normotensive with a heart rate of 110 bpm. WBC was 22,000 cells/uL with bandemia. Sodium was 129 mmol/L, and the rest of the labs were within normal range. Table 1 shows the patient's lab findings compared to normal adult findings.

**Table 1:** Laboratory normal values compared to patient lab values

Laboratory Parameter	Reference Interval	Patient Results
Temperature (Celsius)	36.5	38
Heart Rate (bpm)	60-100	110
Serum Sodium (mmol/L)	135-145	129
White Blood Cell count (/uL)	4,500-11,000	22,000
Bands (x10^9^/L)	0-5	5 (bandemia)

There was extensive scarring to his body, including his face. There was contracture to his elbow joints with only 45 degrees extension capability.

The left lower extremity revealed scarring from skin grafting extending circumferentially distal to the patella. At the anterior and lateral aspect of the leg, there was a large exophytic lesion measuring 20 x 22 cm with edema, spontaneous purulent drainage at various aspects within the wound. There were friable, caseous areas surrounding the areas of purulence. There were palpable, mobile, non-tender lymphadenopathies in the left inguinal region.

The lower right extremity was also notable for extensive scarring at the skin-grafted sites extending distal to the patella. There was minimal purulent drainage in scattered areas within the scars and less caseous areas than the contralateral leg. No lymphadenopathies were palpable in the right inguinal region. A CT scan of the abdomen and pelvis did not reveal bone involvement.

Given the timeline of the burn scars, a presumptive diagnosis of Marjolin’s ulcer of the left leg was made with a differential diagnosis of necrotizing soft tissue infection. He was started on broad-spectrum intravenous antibiotics. The patient was taken to the OR for incision and drainage, excisional debridement down to the fascia, and wound washout. A second look operation was performed 24 hours later, and local excision was additionally performed to achieve a 3 cms circumferential margin of normal tissue.

The pathological tissue diagnosis of squamous cell carcinoma, identified by infiltrating nests and cords of tumor cells with multifocal keratinization, concurred with the clinical presumptive diagnosis. The following are photographs of the tissue submitted at the time of surgery.

**Figure 1:**
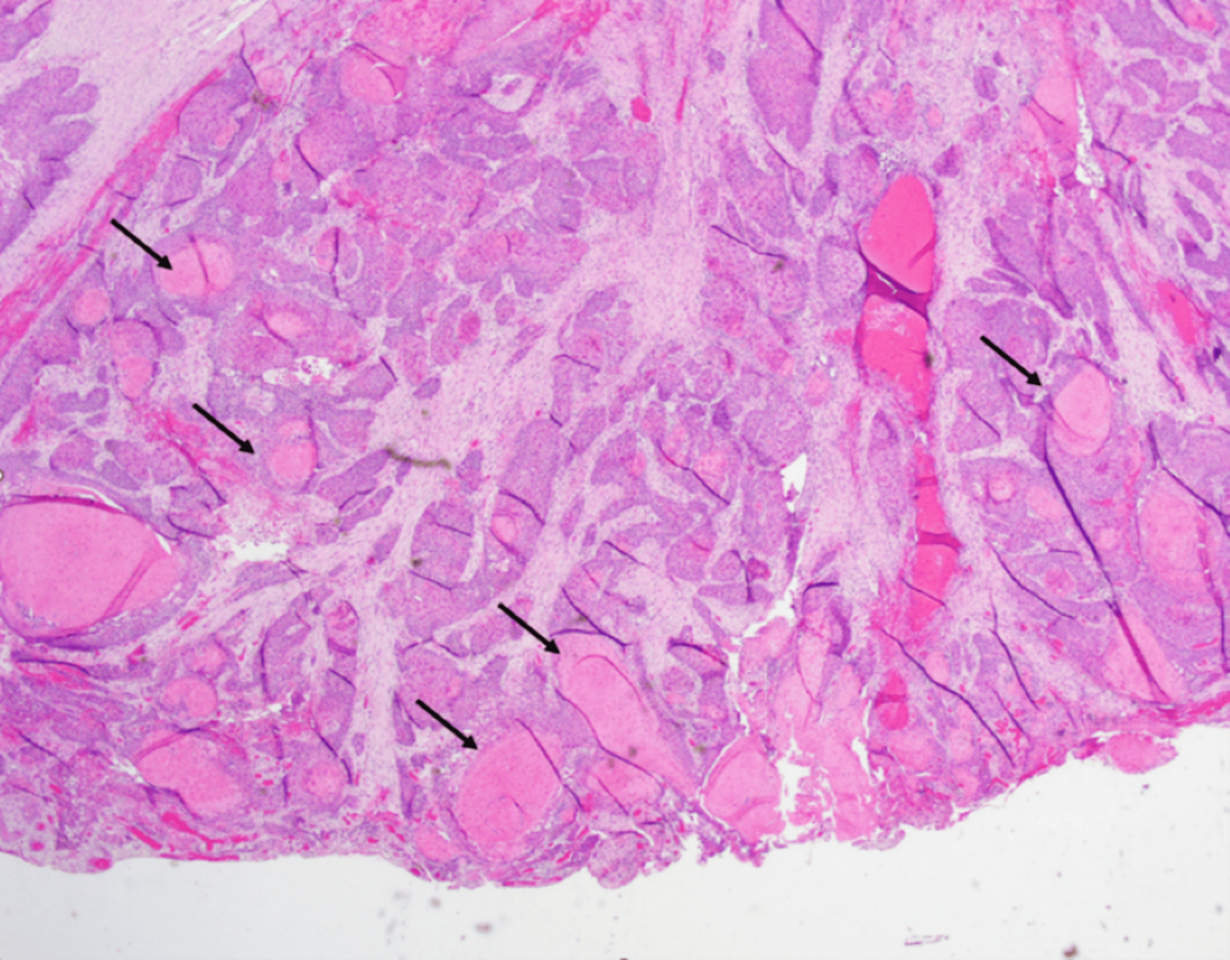
H&E stain: Low power view (original magnification x10) reveals infiltrating nests and cords of tumor cells with multifocal keratinization (arrows).

Figure 1 depicts areas of the submitted tissue showing cords of tumor cells on low power field indicated by arrows.

**Figure 2:**
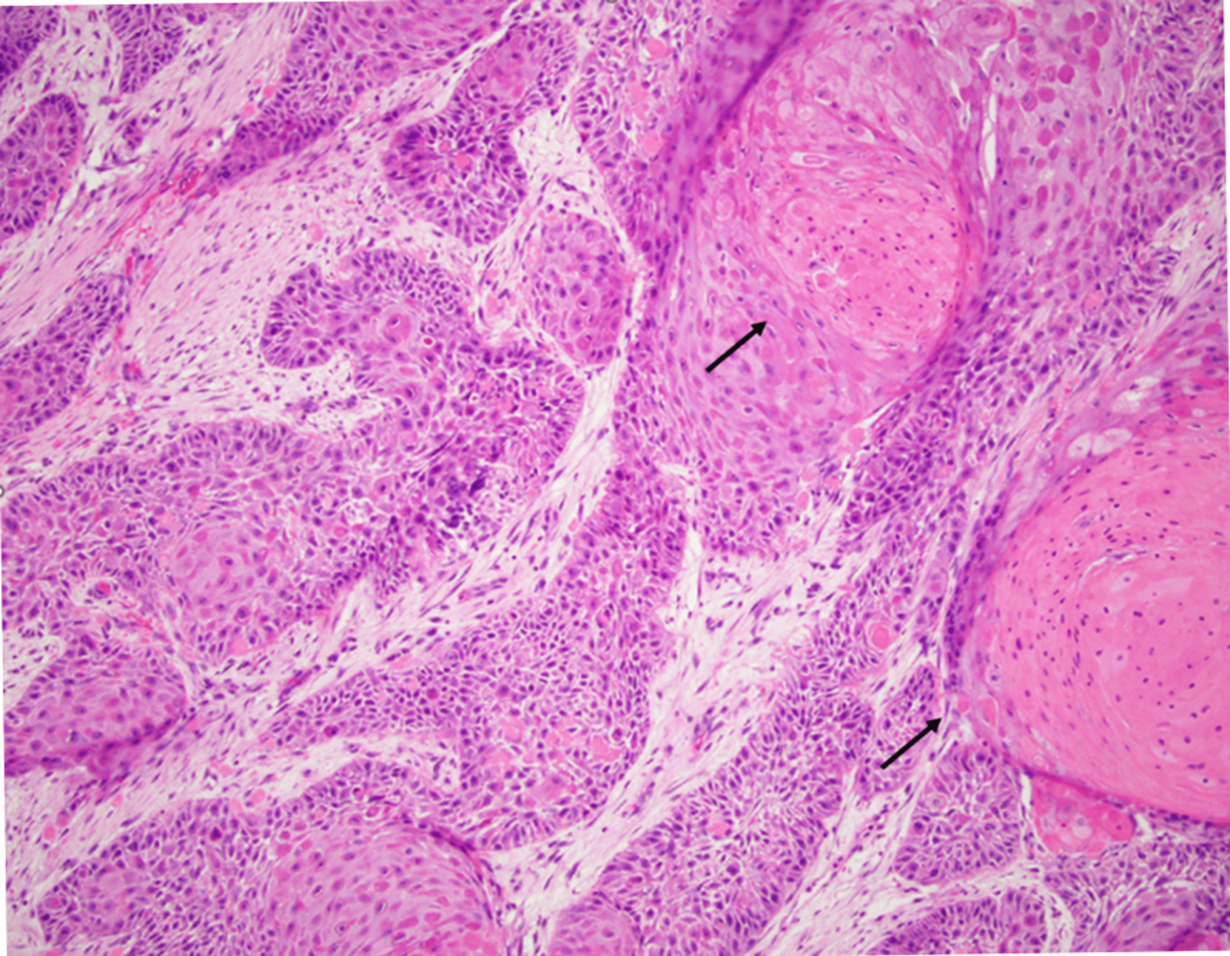
Medium power again reveals the keratinization in the tumor nests, with onion skin type of appearance. In between the tumor nests, desmoplastic stroma is seen.

Figure 2 highlights areas of the submitted tissue on medium power magnification showing keratinized areas of the tumor with intertwining areas of desmoplastic reaction in the stroma.

**Figure 3:**
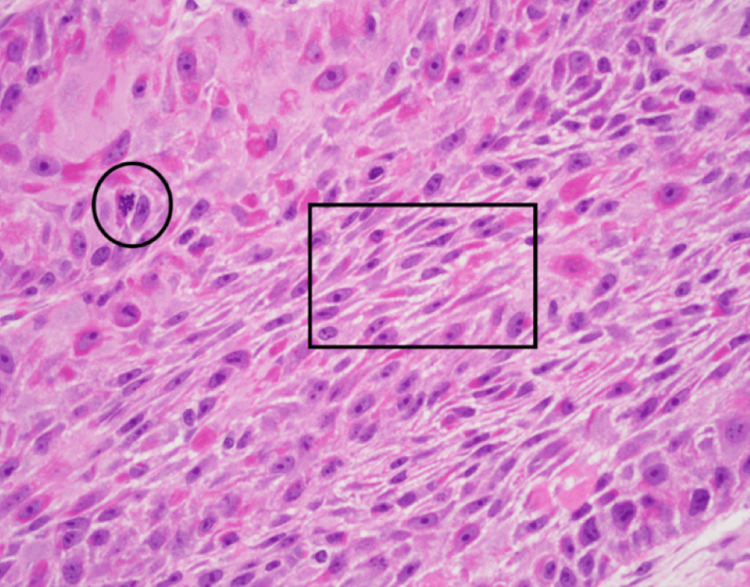
On high power, the cellular pleomorphism is appreciated. There are mitotic figures (circled). Intercellular bridges are seen in the square, supporting squamous differentiation.

High-powered magnification revealed cellular pleomorphism associated with increased mitotic figures and intercellular bridges characteristic of squamous cell differentiation (Figure 3).

Postoperatively, he was managed in a monitored setting. The initially administered broad-spectrum antibiotic therapy with vancomycin, clindamycin, and meropenem to target typical polymicrobes found in the setting of necrotizing soft tissue infection was continued while awaiting wound cultures. Nutritional optimization and supportive care were implemented. He was subsequently transferred to a Burn Center for further management of the wounds.

## Discussion

Marjolin’s ulcer is a rare and aggressive malignancy that arises from chronic nonhealing tissue, most commonly burn scars, but also in other forms of damaged tissue like pressure sores, traumatic wounds, chronic ulcers, and others [3]. While not all burn scars undergo malignant transformation, it is important to note when they do. MU is diagnosed using an incisional biopsy of the suspected area. If the histological specimen has evidence of a cancerous change, an MU may be diagnosed.

The exact pathophysiological mechanisms underlying MU development remain incompletely understood. Theories suggest multifactorial pathogenesis involving injury, the formation of a scar, and the destruction of local blood and lymphatic vessels. This results in an immune-privileged site shielding the tissue from antitumor antibodies, thus allowing malignant transformation to occur [4].

Genetic changes can lead to increased cell proliferation and malignancy. Common mutations include alterations in the TP53 tumor suppressor gene, and activation of oncogenes like KRAS may also contribute to these changes. Loss of heterozygosity (LOH) at chromosome 9p21 has been frequently observed, and mutations in the CDKN2A gene, which regulates the cell cycle, have also been implicated.

Other theories include chronic inflammation and trauma contributing to its pathogenesis. MU has the potential for lymph node and distant metastasis [2], making the diagnosis and treatment of this condition urgent. Squamous cell carcinoma is the most frequent subtype, but other subtypes, such as basal cells or melanoma, have been reported [5]. In this patient, imaging studies, including CT scans of the chest, abdomen, and pelvis, did not reveal distant metastasis, and the palpable inguinal lymph nodes encountered on physical examination revealed inflammatory changes.

Surgical excision with wide margins remains the mainstay of treatment for MU. Recommendations by the National Comprehensive Cancer Network (NCCN) guidelines currently state: low-risk cutaneous SCC: 4-6 mm margins, excision to mid-subcutaneous fat with histological margin assessment, high-risk or very high-risk cutaneous SCC: > 6-10 mm margins. Mohs or margin-controlled excision is usually preferred for high-risk disease. For trunk or extremity SCC as in MU of the lower extremity, recommended margins are >2 cms. In this case, 3 cms circumferential margins were obtained.

Treatment aims to achieve total removal of malignant tissue and minimize recurrence risk [5]. However, in cases with extensive distant and proximal metastasis, the optimal surgical approach is still a topic of debate [6]. The role of adjuvant therapies such as radiation and chemotherapy remains controversial. There is still very limited supporting evidence for their utility in improving outcomes or decreasing recurrence rates [7].

The prognosis of MU is influenced by many factors, including stage, histological subtype, and presence of metastasis [8]. Consistent with previous studies, evidence of advanced-stage disease, presence of proximal or distant metastasis, and high-grade histological findings are associated with a poor prognosis and decreased survival rate [7]. Making early identification and intervention critical to improving the outcomes of patients with MU further emphasizes the importance of surveillance of high-risk patients and prompt intervention [3].

## Conclusions

MU remains a challenging clinical entity due to its variable presentation and aggressive nature. To optimize outcomes in patients with MU, a multidisciplinary approach involving early diagnosis, staging, and individualized treatment is required. Continued research is needed to determine its pathogenesis and explore new treatment options accurately, to better our understanding of this disease, and to improve patient outcomes.

This case underscores the need for a high index of suspicion for malignant transformation in chronic or previously grafted burn scars presenting with infection-like features. Early biopsy remains crucial to prevent diagnostic delay.
